# An optogenetic toolbox of LOV-based photosensitizers for light-driven killing of bacteria

**DOI:** 10.1038/s41598-018-33291-4

**Published:** 2018-10-09

**Authors:** Stephan Endres, Marcus Wingen, Joaquim Torra, Rubén Ruiz-González, Tino Polen, Gabriela Bosio, Nora Lisa Bitzenhofer, Fabienne Hilgers, Thomas Gensch, Santi Nonell, Karl-Erich Jaeger, Thomas Drepper

**Affiliations:** 1 Institute of Molecular Enzyme Technology, Heinrich-Heine-University Düsseldorf, Forschungszentrum Jülich GmbH, Jülich, Germany; 2 m2p-labs GmbH, Baesweiler, Germany; 3 Institut Químic de Sarrià, Universitat Ramon Llull, Barcelona, Spain; 4 Institute of Bio- and Geosciences, IBG-1: Biotechnology, Forschungszentrum Jülich GmbH, Jülich, Germany; 5 Instituto de Investigaciones Teóricas y Aplicadas, Universidad Nacional de La Plata, La Plata, Argentina; 6 Institute of Complex Systems, ICS-4: Cellular Biophysics, Forschungszentrum Jülich GmbH, Jülich, Germany

## Abstract

Flavin-binding fluorescent proteins (FPs) are genetically encoded *in vivo* reporters, which are derived from microbial and plant LOV photoreceptors. In this study, we comparatively analyzed ROS formation and light-driven antimicrobial efficacy of eleven LOV-based FPs. In particular, we determined singlet oxygen (^1^O_2_) quantum yields and superoxide photosensitization activities via spectroscopic assays and performed cell toxicity experiments in *E*. *coli*. Besides miniSOG and SOPP, which have been engineered to generate ^1^O_2_, all of the other tested flavoproteins were able to produce singlet oxygen and/or hydrogen peroxide but exhibited remarkable differences in ROS selectivity and yield. Accordingly, most LOV-FPs are potent photosensitizers, which can be used for light-controlled killing of bacteria. Furthermore, the two variants Pp2FbFP and DsFbFP M49I, exhibiting preferential photosensitization of singlet oxygen or singlet oxygen and superoxide, respectively, were shown to be new tools for studying specific ROS-induced cell signaling processes. The tested LOV-FPs thus further expand the toolbox of optogenetic sensitizers usable for a broad spectrum of microbiological and biomedical applications.

## Introduction

Antimicrobial photodynamic inactivation (aPDI) and photodynamic therapy (PDT) have been developed and applied for treating localized microbial infections and solid tumors (for example see references^1–3^). Both therapies are principally based on the local and light-driven formation of cytotoxic reactive oxygen species (ROS), which immediately induce cell damage during illumination. Here, ROS formation specifically relies on the combination of (i) a phototoxic, light-absorbing compound referred to as photosensitizer (PS), (ii) an excitation source, emitting light of the appropriate wavelength that can be absorbed by the PS and finally (iii) molecular oxygen (O_2_). After light absorption, the PS undergoes a transition from the electronic ground state to a singlet excited state and, via intersystem crossing, further to its triplet excited state. The long lifetime of the PS triplet state allows it to produce ROS through either energy transfer to O_2_ yielding singlet oxygen (^1^O_2_; type-II mechanism) or through electron transfer from a neighborhood donor to produce a radical anion that further reacts with O_2_ to generate primarily the superoxide radical anion (O_2_^•−^) and, through a cascade of redox reactions, other ROS such as hydrogen peroxide (H_2_O_2_) and hydroxyl radicals (HO^•^). This pathway is referred to as the type-I mechanism. Most PSs are capable of undergoing both type-I and -II reactions whereby the outcome of the competition being strongly conditioned by the PS micro-environment. Intracellular ROS production, in turn, results in rapid photo-oxidation of different macromolecules, including proteins, membrane lipids, as well as DNA and RNA, which can finally lead to cell death^4,5^. In particular, ^1^O_2_ is highly reactive and has a lifetime of about 3 µs and a limited diffusion range of approximately 270 nm^6,7^ in a cell, making it a potent toxic agent for aPDI and PDT^8^.

Exogenously applied dyes have several limitations as PS, including their poor selectivity and limited pharmacokinetics. Therefore, alternative PS with better selectivity towards bacteria and tumor tissue and higher cell-killing efficiency need to be developed. Genetically encoded photosensitizers (i.e. proteins that bind - covalently or non-covalently - a chromophore capable of ROS photosensitization) are a new class of PS that exhibits many advantages for different biological and medical applications: intracellular synthesis and accumulation can be controlled and adapted by using inducible expression systems^9^. In addition, the PS can be fused to specific targeting sequences (e.g. leader peptides or antibodies) to selectively direct the recombinant protein to particular cellular structures, compartments or cell types of interest.

**Figure 1:**
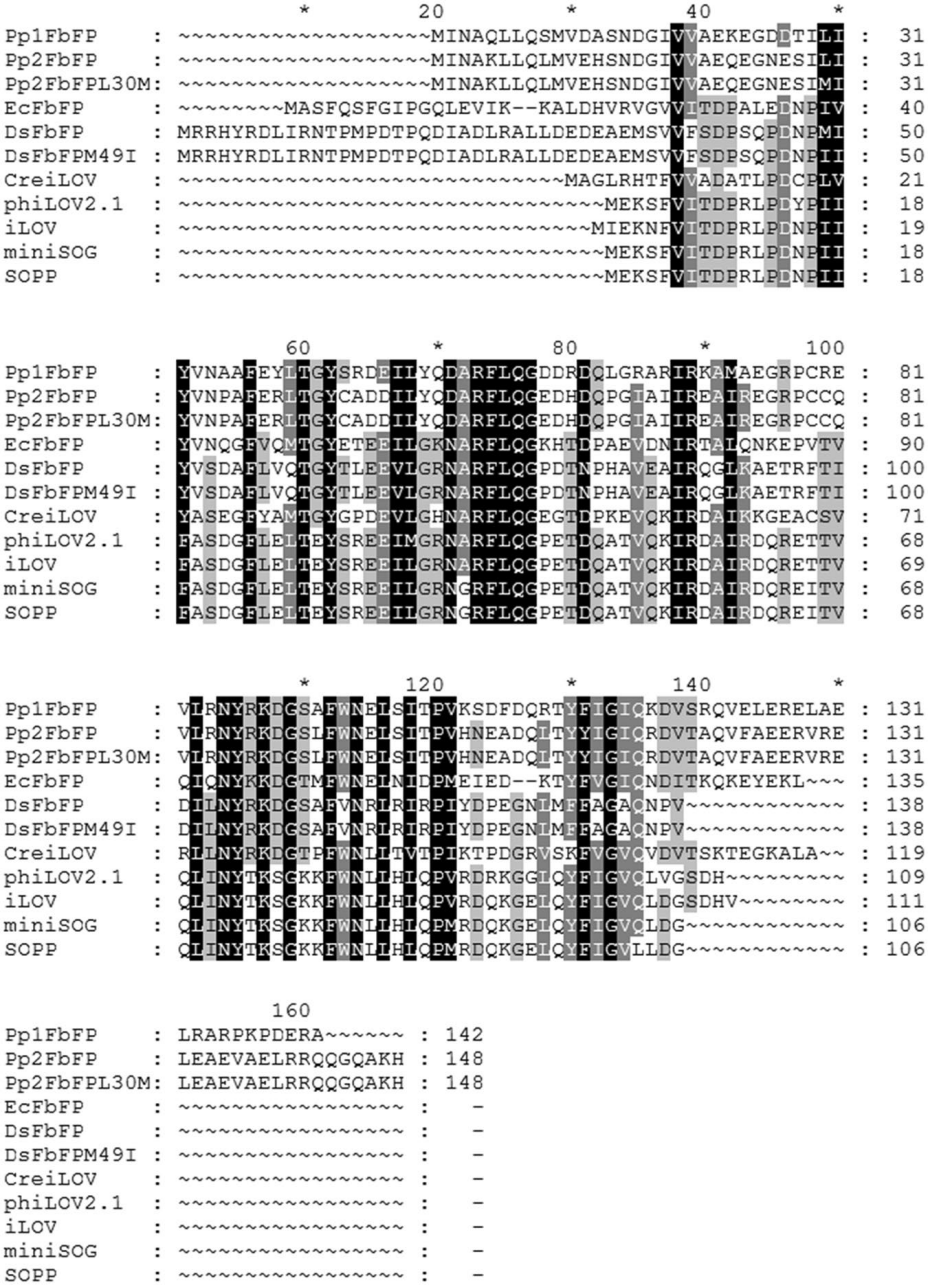
Multiple amino acid sequence alignment of the tested LOV-based fluorescent proteins. Homologous and similar residues are marked in black and grey.

In the last decade, two different classes of optogenetic sensitizers have been developed. The first class encompasses fluorescent proteins of the green fluorescent protein (GFP) family^10,11^, whereas the second class harbors flavin-binding fluorescent proteins that are derived from light-oxygen-voltage (LOV) photoreceptor domains^12–14^.

Photosensitizers of the GFP family: First studies demonstrated that GFP is a rather ineffective photosensitizer, whose ROS-production efficiency is influenced by the chromophore’s accessibility to molecular oxygen^11,15,16^. In contrast, KillerRed, a dimeric GFP-homolog derived from the non-fluorescent hydrozoan chromoprotein anm2CP, was shown to be an efficient photosensitizer that primarily generates O_2_^•−^ via type-I reaction^10,17,18^. This photosensitizer was successfully applied for photo-inducible killing of targeted cell populations^10,19–22^, directed inactivation of proteins via chromophore assisted light inactivation (CALI)^23–26^ and ROS-signaling^27,28^. Recently, the monomeric KillerRed derivatives such as SuperNova and KillerOrange have been developed, which exhibit phototoxicity in bacteria and mammalian cells and further allow fusion to target proteins for CALI without affecting the quaternary structure^29–31^. Structural analyses revealed that a water-filled channel connecting the chromophore with the protein surface might lead to an increased photosensitizing activity via facilitated O_2_ and ROS transport^31,32^. In contrast to the above mentioned GFP-based PS, the red monomeric protein TagRFP, derived from the *Entacmaea quadricolor* fluorescent protein TurboRFP^33^ generates ^1^O_2_^16^ and was shown to kill *E*. *coli* by endogenously generated ^1^O_2_ upon green light irradiation^34^.

**Figure 2:**
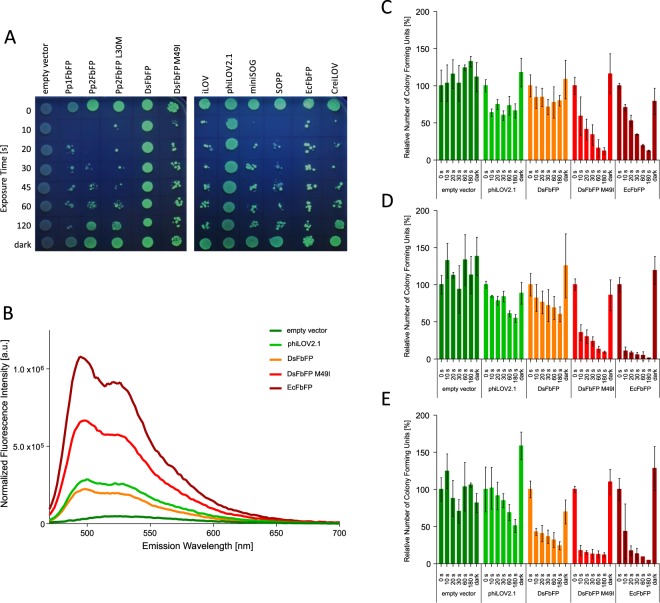
*In vivo* phototoxicity assay of LOV-based fluorescent proteins. (**A**) Plate spot assay. Samples of *E*. *coli* cultures expressing the respective FP, were illuminated with blue light (λ = 448 nm; 130 mW cm^−^²) for a defined period of time and subsequently placed on agar plates. Colony growth in dependence of the illumination time served as a first indicator for individual LOV-FP phototoxicities. The empty vector and samples of each culture that were kept in the dark were used as controls. Green colonies represent fluorescing cells while colonies of non-fluorescing cells appear blueish due to UV-A-light illumination. (**B**–**E**) Analysis of colony forming units (CFU). The colony forming capacity of FbFP-expressing *E*. *coli* BL21 (DE3) cells was investigated after 0, 10, 20, 30, 60 and 180 s of blue light irradiation. Samples of *E*. *coli* cells harboring the respective FbFP expression plasmids were incubated for three hours after induction and fluorescence emission spectra of the respective cell extracts were measured in PBS buffer (**B**). Fluorescence spectra of the five bacterial cultures were normalized to their cell density characterized by the absorbance at 580 nm (OD_580_). For CFU determination, cell cultures were diluted to a final cell density of OD_580_ = 0.1 in PBS buffer (pH 7.4). Subsequently, cells were illuminated using different intensities of blue light (10 mW cm^−2^ (**C**); 90 mW cm^−2^ (**D**) and 130 mW cm^−2^ (**E**)). At given time points, aliquots of the irradiated cells were transferred to LB agar plates and incubated overnight at 37 °C in the dark. The data represents the mean values of three independent experiments and standard deviations are indicated by error bars.

Photosensitizers of the LOV-FP family: Recently, efforts to produce genetically encoded fluorescent proteins that efficiently generate intracellular ^1^O_2_ for correlative light electron microscopy (CLEM) have turned to the generation of miniSOG (mini Singlet Oxygen Generator) engineered from the *Arabidopsis thaliana* phototropin 2 LOV2 domain (*At* LOV2 phot2)^35^. Photophysical properties of miniSOG were extensively characteriezed^35–37^ and the photosensitizer has been successfully applied as tag for CLEM^35,38–45^, cell ablation in *Caenorhabditis elegans* and *Drosophila melanogaster*^46–49^ as well as for light-induced killing of targeted cancer cells^50,51^. Since then a large number of miniSOG derivatives has been developed, including SOPP (singlet oxygen photosensitizing protein)^52^, miniSOG Q103V^45^, miniSOG2^49^, and SOPP3^53^. While previous works have focused on characterizing and improving miniSOG’s properties, comparatively less effort has been devoted to explore other LOV-FPs from different origins. In this work, we therefore comparatively analyzed eleven LOV-FPs derived from microbes and plants with a focus on the antimicrobial phototoxicity and their ability to produce ROS via type-I and type-II reactions.

**Figure 3:**
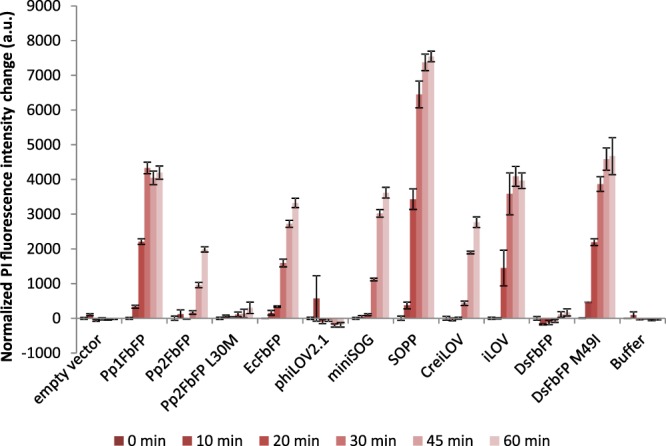
Analysis of LOV-FP phototoxicity using propidium iodide as quantitative marker for dead *E*. *coli* cells. The bars show the change in PI fluorescence intensity (λ_ex_ = 535 nm, λ_em_ = 617 nm) over 60 minutes of blue-light illumination (~10 mW cm^−^²). The data was normalized to the amount of functional protein per cell. The corresponding raw data can be found in Supplemental Fig. S1. To ascribe the observed effects to blue-light exposure, a control experiment in the dark was conducted, which showed no significant changes for all tested LOV-PS (Supplemental Fig. S2). The data represent the mean values of three independent experiments, the error bars indicate the calculated standard deviations.

LOV-FPs have been originally designed as alternative FPs that can be used for *in vivo* analysis of cellular functions in the absence of molecular oxygen^54,55^. The first variants of this new class of *in vivo* reporter proteins were either derived from (i) bacterial photoreceptors, including proteins from *Pseudomonas putida*, *Dinoroseobacter shibae* and *Bacillus subtilis*, termed as FMN-binding fluorescent proteins (FbFPs) or (ii) *Arabidopsis thaliana* phototropin2 LOV2 domain, designated as iLOV^56^ and its more photostable variant phiLOV^57^. In addition, new LOV-based FPs have recently been developed by engineering photoreceptors from *Chlamydomonas reinhardtii* (CreiLOV)^58^ and thermophilic microbes^59^. Many of the LOV-FPs have been successfully applied as intracellular reporters in different pro- and eukaryotic cells under hypoxic and anoxic conditions^54,60–64^. Moreover, a detailed overview of the photophysical characteristics of several LOV-FPs was recently published^65^.

In this study, we now could show that most of the tested LOV-FPs are potent photosensitizers that can be used for efficient killing of microbial cells and for studying ROS-induced stress responses in a light-dependent manner.

## Results

### *In vivo* assessment of LOV-FP-mediated phototoxicity in *E*. *coli*

Within the last ten years, many different flavin-binding fluorescent proteins that are based on LOV photoreceptor proteins from plant, algae and bacteria have been successfully established and applied^55,66^. Among them, the two LOV-FP derivatives miniSOG and SOPP were initially engineered as potent, genetically encoded photosensitizers that are capable of producing sufficient amounts of ROS (predominantly ^1^O_2_) for targeted cell killing^35–37,48,50–52^. All of the phototoxicity studies, however, have been carried out in nematodes or cancer cells but not in bacteria, the primary target in aPDI. To this end, we first comparatively analyzed the light-induced antimicrobial activities of eleven LOV-FPs, exhibiting moderate to high sequence identities but different fluorescence quantum yields (*Φ*_F_) ranging from 0.20 (phiLOV2.1) to 0.44 (EcFbFP) (Fig. 1 and Table 1). Besides miniSOG and SOPP, the LOV-FPs iLOV and phiLOV2.1 are derived from the *A*. *thaliana* Phot2 LOV2 domain^35,52,56,57^. In contrast, the other seven representatives of the LOV-FP family are based on microbial LOV photoreceptors from *P*. *putida* (Pp1FbFP, Pp2FbFP, Pp2FbFP L30M^54,65,67^), *D*. *shibae* (DsFbFP, DsFbFP M49I^65^,), *B*. *subtilis* (EcFbFP^54^) and *C*. *rheinhardtii* (CreiLOV^58^) of which Pp2FbFP L30M was recently reported to efficiently produce ^1^O_2_ upon blue light irradiation^67^. To get a first impression of the potential for aPDI, we initially compared the phototoxicity of LOV-FPs in *E*. *coli* BL21 (DE3) cells during illumination with intense blue light (λ_max_ = 448 nm, 130 mW cm^−^²) using a simple plate spot assay (Fig. 2A). Approximately half of the tested LOV-FPs exhibited strong light-triggered antimicrobial activities, resulting in a pronounced growth impairment, already after only 10 seconds of blue-light irradiation. Among the other, Pp2FbFP, Pp2FbFP L30M or CreiLOV required longer illumination, whereas *E*. *coli* cells expressing phiLOV2.1 or DsFbFP were almost unaffected even after prolonged exposure to blue-light.

**Table 1:** Singlet oxygen quantum yields (*Φ*_Δ_) and photophysical properties of LOV- and GFP-based photosensitizers.

Name	Source organism	Excitation λ_max_ (nm)	Emission λ_max_ (nm)	*ε* (M^−1^ cm^−1^)	*Φ*_F_	Brightness (M^−1^ cm^−1^)	*Φ*_Δ_
**Pp1FbFP**	*P*. *putida*	450^65^	496^65^	13,900 ± 500^65^	0.27 ± 0.01^65^	3,750^65^	0.23
**Pp2FbFP**	*P*. *putida*	449^65^	495^65^	14,200 ± 50^65^	0.22 ± 0.01^65^	3,120^65^	0.11
**Pp2FbFP L30M**	*P*. *putida*	449	495	14,800 ± 100	0.25 ± 0.01	3,700	0.10
**DsFbFP**	*D*. *shibae*	449^65^	498^65^	14,300 ± 50^65^	0.35 ± 0.01^65^	5,000^65^	0.33
**DsFbFP M49I**	*D*. *shibae*	450	498	13,700 ± 500	0.36 ± 0.01	4,930	0.42
**EcFbFP**	*B*. *subtilis*	448^65^	496^65^	14,500 ± 200^65^	0.44 ± 0.01^65^	6,380^65^	0.07
**iLOV**	*A*. *thaliana*	450^94^	497^94^	14,800 ± 300^94^	0.33 ± 0.01^94^	4,850	0.05
**phiLOV2.1**	*A*. *thaliana*	450^65^	497^65^	n.d. ^65^	0.20 ± 0.01^65^	2,840	0.01
**miniSOG**	*A*. *thaliana*	447^65^	497^65^	14,200 ± 700^65^	0.41 ± 0.01^65^	5,820^65^	0.03^36,37^ 0.04^this study^
**SOPP**	*A*. *thaliana*	440	490	n.d.	0.33 ± 0.01	4,690	0.25
**CreiLOV**	*C*. *reinhardtii*	449	497	14,200 ± 400	0.32 ± 0.01	4,540	0.04
***miniSOG Q130V***	*A*. *thaliana*	n.p.	n.p.	n.p.	n.p.	n.d.	0.39^45^
***SOPP3***	*A*. *thaliana*	439^53^	490^53^	15,000^53^	0.41^53^	6,150	0.60^53^
***TagRFP***	*E*. *quadricolor*	555^33^	584^33^	100,000^33^	0.48^33^	48,000	0.004^16^
FMN		444^65^	531^65^	12,200^95^	0.25 ± 0.01	3,050	0.57

Extinction coefficient (ε) and fluorescence quantum yield (*Φ*_F_) of LOV-based PS that were analyzed in this study (highlighted in bold) are given as mean values with standard deviations determined from three independent measurements. The fluorescence brightness is the product of individual ε and *Φ*_F_ values. The extinction coefficient as well as the brightness of phiLOV2.1 and SOPP could not be determined directly (n.d.), as both proteins aggregated at 95 °C. Therefore, for further calculations, an average extinction coefficient of 14,200 M^−1^cm^−1^ was assumed for these variants. For a better comparability, values of miniSOG Q103V, SOPP3 and TagRFP (marked in bold and italic) that have been obtained from indicated publications were also listed; n.p.: not published.

To further characterize LOV-FP mediated phototoxicity, we quantitatively determined the cell viability by counting colony forming units, which is more sensitive due to sample dilution before spreading light-treated cells onto agar plates. The viability of *E*. *coli* cells expressing representative FbFPs that exhibited either low (phiLOV2.1 and DsFbFP) or high (DsFbFPM49I and EcFbFP) activity in the plate spot assay was measured in dependence on exposure time and applied light intensity. As shown in Fig. 2C–E, increasing the illumination time (10–180 seconds), as well as the light intensity (10, 90 and 130 mW cm^−2^), gradually decreased the number of viable bacteria for all FbFPs, although for phiLOV2.1 and DsFbFP cell death was more modest (Fig. 2E). In contrast, in the case of DsFbFP M49I and EcFbFP (these variants already showed pronounced phototoxicities in the plate spot assay) almost all cells were killed under these illumination conditions. Analysis of LOV-FP-mediated fluorescence emission in corresponding cell extracts revealed that fluorescence intensities (and thus protein accumulation levels) differed strongly for the tested *E*. *coli* strains (Fig. 2B). Thus, a better comparison of LOV-FPs should take into account such differences in protein levels. To this end, we comparatively analyzed individual LOV-FP phototoxicities using the propidium iodide cell-death assay^68^ and normalized it to the respective *in vivo* fluorescence (see materials and methods for details). To precisely determine differences in phototoxic activities, we applied low light intensities (10 mW cm^−2^) in this assay. The results presented in Fig. 3 show the increase in PI fluorescence in dependence of illumination time (0 to 60 minutes).

Based on the normalized PI fluorescence, *in vivo* phototoxicity of the tested LOV-FP-based photosensitizers can be classified into three different groups: The first encompasses SOPP, DsFbFP M49I, Pp1FbFP and iLOV, where blue-light illumination of *E*. *coli* cells resulted in a fast and strong increase of PI fluorescence. Thus this group of LOV-FPs exhibits a high, light-triggered antimicrobial activity in our assay. The second group contains the medium-toxic variants Pp2FbFP, EcFbFP, miniSOG and CreiLOV, whose PI fluorescence signals developed slower during blue-light illumination and exhibited a lower intensity compared to those of the first group. The third LOV-FP group contains the less-toxic proteins phiLOV2.1, DsFbFP and Pp2FbFP L30M. Samples of *E*. *coli* cells expressing these three variants did not show a significant increase in PI fluorescence upon the applied low-light conditions. In a control experiment, where aliquots of the same *E*. *coli* LOV-FP expression cultures were kept in the dark for 60 minutes, no significant changes in the PI fluorescence could be detected (Supplemental Fig. S2), thereby confirming that the observed increase in PI fluorescence is specifically induced by the phototoxic activity of the tested LOV-FPs.

While the PI-based experiment presented in Fig. 3 indicates that most LOV-FPs can damage the cell envelope of *E*. *coli* via light-induced ROS formation, it is not clear whether this is – at the level of single cells – an antimicrobial effect that occurs homogeneously within the culture. To demonstrate that the increase of PI fluorescence directly correlates to the growing number of killed cells under continuous blue-light illumination (in contrast to the possibility, that just a subset of susceptible bacteria is responsible for the PI fluorescence signal due to an individually increasing level of mRNA under the tested conditions), development of the PI signal was analyzed in single cells using a fluorescence microscope. As shown in Supplemental Fig. S3, *E*. *coli* cells expressing either DsFbFP (Supplemental Fig. S3A) or DsFbFP M49I (Supplemental Fig. S3B) exhibited a detectable FbFP-mediated green fluorescence (consider images I and II) as well as a homogeneous development of a strong PI fluorescence as a function of time (consider images III-VIII) after illumination (102 mW; 460–490 nm). The corresponding control (*E*. *coli* cells carrying the corresponding empty expression vector) did not show a detectable PI signal even after prolonged blue-light exposure times (Supplemental Fig. S3C). Furthermore, as expected from *in vivo* phototoxicity assays, the two investigated LOV-FPs exhibit rather different phototoxicities: While expression of DsFbFP M49I resulted in maximal, saturating PI fluorescence signal after 105 s, the PI fluorescence signal of DsFbFP accumulating cells was significantly lower even after longer blue light illumination. The microscopic analysis thus clearly demonstrates that (i) illumination of LOV-FPs accumulating *E*. *coli* cells results in a homogeneous increase of PI influx (and thus increase of PI fluorescence), which shows (ii) a clear dependency on illumination time and (iii) most probably on individual properties of tested LOV-FPs.

**Figure 4:**
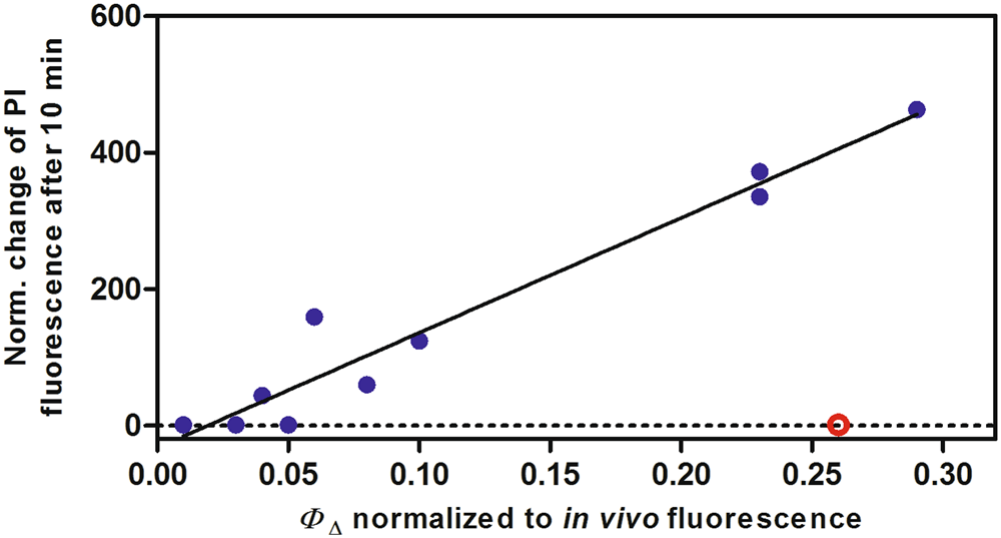
Protein phototoxicity in *E*. *coli* cells in relation to singlet oxygen production. Correlation between the extent of cell death, measured by propidium iodide (PI) fluorescence, and the amount of singlet oxygen produced during the first 10-min irradiation period. This was calculated based on multiplying the singlet oxygen quantum yield by the percentage of protein fluorescence remaining after 10-min irradiation. The underlying reason for this correction is that the proteins photobleach to different extent over time (see Supplemental Fig. S4, Fig. 3 and ref.^65^), hence the rate of singlet oxygen production during the irradiation period considered has decreased concomitantly. The protein fluorescence is a measure of the amount of protein still intact after the irradiation period. The data point indicated by the red circle represents the determined values of the outlier DsFbFP.

In summary, our *in vivo* data clearly demonstrate that many of the tested LOV-FPs can be used as photosensitizers suitable for efficient light-controlled killing of *E*. *coli* cells. These properties thus render them potentially suitable for antimicrobial photodynamic inactivation (aPDI). In comparison to conventional treatment of bacterial infections with antibiotics, aPDI exhibits several advantages, including a ROS-based broad spectrum of activities against a multitude of different microbes together with a high efficacy towards resting cells and pathogenic strains that exhibit multiple antibiotic resistances (e.g. reviewed in refs^69,70^). Furthermore, because of the high reactivity and short lifetime of ROS and the resulting damage of very different essential biomolecules in the targeted microbe, formation of resistances to aPDI in bacteria must be considered highly unlikely even when cells are treated repetitively with sublethal doses of light^71–73^. In addition to that, the possibility of controlling the expression of FbFPs in the pathogenic bacteria offers an added attractive to this new class of biological photosensitizers.

### *In vitro* measurements of LOV-FP-dependent singlet oxygen production

In order to analyze, if the observed difference in *in vivo* phototoxicities is caused by distinct ^1^O_2_ production levels, we next determined individual singlet oxygen quantum yields of the purified fluorescent proteins by measuring the ^1^O_2_ phosphorescence at λ = 1275 nm in deuterated PBS buffer as described in Torra *et al*.^67^. The results of these experiments are summarized in Table 1 and show a broad variability of ^1^O_2_ photosensitization yields. DsFbFP M49I exhibited the highest ^1^O_2_ *Φ*_Δ_ with 0.42. Furthermore, the value for SOPP (*Φ*_Δ_ = 0.25) matches the published value from Westberg *et al*., who generated this protein by means of site-directed protein engineering based on miniSOG^52^. These two values also correlate well with the high phototoxicity *in vivo*. The *Φ*_Δ_ values of the other LOV-FPs are significantly smaller, ranging from 0.01 (phiLOV2.1) to 0.33 (DsFbFP). The lowest singlet oxygen quantum yield of phiLOV2.1 is also in good agreement with the observed absence of *in vivo* toxicity. In addition, some of the LOV-FPs with intermediate singlet oxygen production rates (e.g. EcFbFP with a *Φ*_Δ_ of 0.07) were also classified as moderately toxic *in vivo* photosensitizers. However, considerable deviations of *in vivo* phototoxicity and ^1^O_2_ quantum yield were also observed for some of the tested LOV-FPs. For example, iLOV illumination resulted in a very strong PI signal in the *in vivo* toxicity assay, but spectroscopic analysis revealed a low *Φ*_Δ_ value (0.05). On the other hand, the high *Φ*_Δ_ value of DsFbFP (0.33) did not correspond to its weak *in vivo* phototoxicity. The discrepancies observed for some of the LOV-based PS were to be expected. Regarding the *Φ*_Δ_ measurements, it is arguable whether in all cases the values derived from solution measurements are representative of the *Φ*_Δ_ values inside the cells. Specifically, the microenvironment of the protein inside a living cell may affect its ability to produce ^1^O_2_ e.g., because of different protein conformations and/or interactions with other cellular components. For example, Westberg *et al*. recently observed that the increase of temperature has a remarkable effect on O_2_-dependent quenching of FMN triplet state – a phenomenon that can also be differently affected by the individual properties of the surrounding protein as described for miniSOG and SOPP^53^. In addition, it must be kept in mind that the *Φ*_Δ_ values are assumed to be proportional to the intensity of ^1^O_2_ phosphorescence, whereas the biological effects are due to ^1^O_2_ molecules being released from the protein. Thus, our measured *Φ*_Δ_ values describe the total amount of ^1^O_2_ molecules produced by the individual photosensitizer, but may not reflect the number of ^1^O_2_ molecules being actually released. This is particularly the case for LOV-FPs with triplet lifetimes of hundreds of microseconds in air-saturated solutions, such as DsFbFP and DsFbFP M49I (*τ*_T_ > 500 μs), which clearly reflects a very low accessibility of molecular oxygen to the chromophore. It is thus reasonable to expect that the generated ^1^O_2_ will only partially escape from the protein. To address this question, indirect ^1^O_2_ detection measurements for the two DsFbFP variants were performed using the chemical trap uric acid (UA), which specifically and irreversibly reacts with ^1^O_2_^74^. The *Φ*_∆_ values determined in dPBS solution were 0.28 and 0.36 for DsFbFP and DsFbFP M49I, respectively, in good agreement with the values obtained by the direct ^1^O_2_ luminescence detection method. These results thus confirm that ^1^O_2_ molecules indeed escape from the protein matrix and reach the bulk solvent. Therefore, the discrepancies observed between ^1^O_2_ production and photokilling experiments should arise from processes other than protein deactivation of ^1^O_2_ molecules.

Returning to the PI results presented in Fig. 3, it is highly revealing that the time evolution of the PI fluorescence leveled off at different upper-limit values for each protein, resulting in a clear sigmoidal shape. This observation is particularly relevant since it implies that photobleaching of the proteins is a critical factor that limits their phototoxic properties. In fact, if the extent of cell death is plotted against the actual *amount* of singlet oxygen produced by each protein during the first 10-min irradiation period, a clear correlation is obtained (Fig. 4), indicating that singlet oxygen is the major cytotoxic species for most LOV-FPs studied and confirming the importance of photobleaching.

### *In vitro* analysis of LOV-FP-catalyzed hydrogen peroxide production

**Figure 5:**
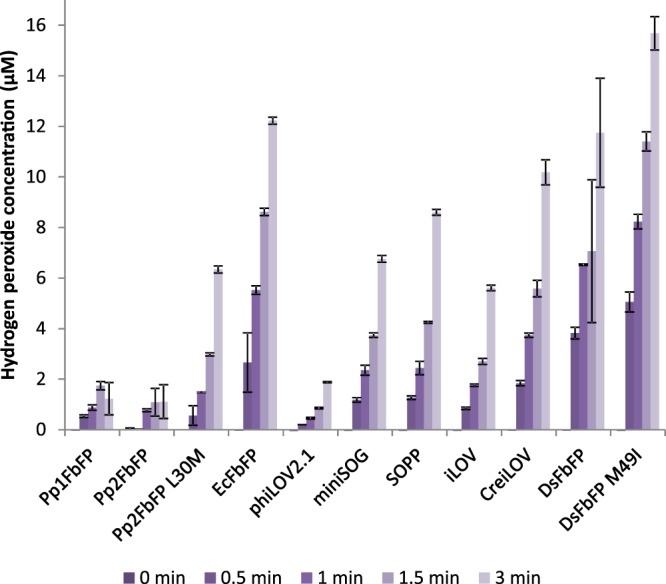
Quantification of LOV-PS-catalyzed hydrogen peroxide formation. The determination of hydrogen peroxide, produced by the LOV-PS as a reaction of blue-light exposure (λ_max_ = 447 nm, ~10 mW cm^−2^), was performed with purified proteins (final concentration OD_450_ = 0.05) by applying the Amplex® Red Hydrogen Peroxide/Peroxidase Assay Kit. The bars show the time-dependent increase of hydrogen peroxide formation for all tested photosensitizes. The control experiment in the dark did not lead to detectable H_2_O_2_ production for all tested proteins (Supplemental Fig. S2). The data represent the mean values of three independent experiments, the error bars the calculated standard deviations.

We further investigated the LOV-FP-dependent production of O_2_^•−^ and H_2_O_2_ that has already been demonstrated for miniSOG and SOPP^37,52^. To this end, we illuminated samples of the purified photosensitizer proteins (final OD_450_ = 0.05) with blue light and analyzed the resorufin-specific fluorescence - the product of the Amplex Red reaction with H_2_O_2_ as described in the material and methods section. The results of this experiment indicate large differences in the H_2_O_2_ production rates of the studied LOV-FPs (Fig. 5). In particular, phiLOV2.1, Pp1- and Pp2FbFP produce low H_2_O_2_ concentrations (≤2 µM), whereas all other tested proteins reached values between ~6 µM and ~16 µM. Especially the two DsFbFP variants as well as EcFbFP and CreiLOV showed notably high hydrogen peroxide production during illumination. The correlation of light treatment and H_2_O_2_ production was evidenced by the dark control which resulted in no significant H_2_O_2_ accumulation in all tested samples (Supplemental Fig. S2). In summary, our data confirmed considerable type-I-driven ROS formation of LOV-FPs, whereas the efficiencies strongly differ between the tested proteins. In this context it is worth mentioning that some of the tested LOV-FPs seem to preferentially produce ROS either via the type-I or type-II photosensitization process, as observed for Pp1- and Pp2FbFP (intermediate ^1^O_2_ and low H_2_O_2_ production rates) and DsFbFP M49I (high H_2_O_2_ and ^1^O_2_ production rates), which make them promising candidates for studying cell signaling processes that are induced by different reactive oxygen species with high spatiotemporal resolution.

### Using Pp2FbFP and DsFbFP M49I as optogenetic tools for analyzing ROS-induced stress responses in *E*. *coli*

In our previous experiments we could demonstrate considerable production of ROS for almost all of the tested LOV-FPs. However, selectivity and efficiency of light-driven ROS formation strongly vary between those proteins. This observation led us to the question if some of the variants can be utilized as selective and non-invasive intracellular ROS generators, to analyze specific responses to oxidative stress in bacteria. Using a DNA-microarray approach, we comparatively analyzed H_2_O_2_ and ^1^O_2_ induced changes in the transcriptome of *E*. *coli* cells, applying either DsFbFP M49I or Pp2FbFP. As expected, a number of *E*. *coli* genes could be identified whose expression rates were significantly upregulated after illumination (Table 2). Remarkably, 25 genes showed higher expression levels in blue-light exposed *E*. *coli* cells accumulating the singlet oxygen and hydrogen peroxide-producing variant DsFbFP M49I. In bacteria, H_2_O_2_ generally serves as signal molecule that can be recognized by the transcriptional regulator OxyR, which in turn specifically activates genes of the OxyR regulon^75–78^. Accordingly, 14 blue-light induced genes belong to the OxyR regulon (Table 2; marked by an asterisk), including the genes *ahpCF* and *katG* encoding the NADH peroxidase and catalase. Both enzymes are known to predominantly scavenge H_2_O_2_ in bacteria, as for example reviewed in^79^. However, the strongest induction was observed for the *dps* gene (~28-fold), which codes for the “DNA-binding protein from starved cells” (Dps) that is known to protect DNA against ROS-mediated damage in *E*. *coli* and other bacteria^80–83^. Furthermore, most of the *suf* genes are induced via DsFbFP M49I-dependent ROS formation. The SufABCDSE complex functions as an alternative iron-sulfur cluster assembly system which can substitute the housekeeping Isc (FeS cluster assembly) system after its hydrogen peroxide-mediated inactivation^78,84^. In addition, some of the blue-light induced genes not belonging to the OxyR regulon code for enzymes that are known to play a role in redox stress response. For example, AzoR, an FMN-dependent NADH-azoreductase, was shown to be essential for growth of *E*. *coli* in the presence of quinones that facilitate accumulation of ROS via deleterious redox cycling^85^.

**Table 2:** DNA microarray-based, genome-wide analysis of ROS-induced stress response mediated by light-exposed LOV-PS in *E*. *coli*.

Gene	Increased expression (x-fold)	*p*-value	Assigned function
**DsFbFP M49I**			
dps*	27.6	0.002	nucleoid-associated protein^80,81^
*azoR*	13.0	0.002	FMN- and NADH-dependent azoreductase^96,97^
sufB*	12.0	0.008	alternative iron-sulfur cluster assembly system^78,84^
*lacI*	11.2	0.048	transcription repressor^98^
ahpC*	7.5	0.005	NADH peroxidase^79^
sufC*	6.8	0.012	alternative iron-sulfur cluster assembly system^78,84^
sufS*	6.7	0.014	alternative iron-sulfur cluster assembly system^78,84^
sufD*	5.5	0.017	alternative iron-sulfur cluster assembly system^78,84^
*yhaK*	5.4	0.014	bicupin-related protein^86^
*pspA*	5.1	0.004	phage shock protein^99^
*ybiJ*	5.1	0.012	Unknown
*glpE*	4.8	0.003	Sulfurtransferase^100^
*mokB*	4.8	0.031	Predicted regulatory peptide
mntH*	4.7	0.021	Divalent metal ion transporter^101^
ahpF*	4.6	0.014	NADH peroxidase^79^
katG*	4.2	0.015	catalase^79^
grxA*	4.2	0.017	glutaredoxin I^102^
hemH*	4.1	0.020	ferrochelatase^78^
trxC*	4.0	0.009	thioredoxin II^103^
*pspD*	3.8	0.003	phage shock protein^99^
*clpS*	3.8	0.002	Part of ClpAP-protease complex^104^
*pspB*	3.7	0.029	phage shock protein^99^
*pspC*	3.3	0.008	phage shock protein^99^
yaaA*	3.1	0.021	Unknown
sufE*	2.9	0.008	alternative iron-sulfur cluster assembly system^78,84^
**Pp2FbFP**			
*azoR*	7.7	0.023	FMN- and NADH-dependent azoreductase^96,97^
*ybiJ*	6.6	0.034	Unknown
*yhaK*	5.6	0.055	bicupin-related protein^86^
*gntK*	4.0	0.046	gluconokinase^105^

LOV-PS Pp2FbFP and DsFbFP M49I were expressed in *E*. *coli* BL21(DE3) cells and illuminated with blue light (Pp2FbFP: 5 min, DsFbFP: 15 min). Transcriptome profiles were compiled from illuminated samples and compared to non-illuminated controls. In this way, genes were identified that showed a significant increase in their expression level (≥3-fold, *p*-value ≤ 0.05), as a reaction to light exposure. Genes that are induced by OxyR are marked with an asterisk. The data represents the mean values of three independent experiments.

In contrast, only four genes were strongly upregulated in *E*. *coli* cells expressing the singlet oxygen forming Pp2FbFP (Table 2), although this LOV-FP variant exerted clear phototoxicity in our *in vivo* experiments (Figs 2 and 3). Two of these genes, namely the above mentioned *azoR* as well as *yhaK*, were also induced in cells expressing DsFbFP M49I and the respective gene products seem either to be potentially involved in sensing (YhaK)^86^ or compensating (AzoR) oxidative stress in *E*. *coli*.

In photosynthetic bacteria, regulatory processes and mechanisms of ^1^O_2_ defense have been extensively analyzed (e.g. reviewed in^7^). In contrast, specific responses of non-phototrophic bacteria towards singlet oxygen is still poorly understood. Kim and coworkers presented first evidences that OxyR might also (directly or indirectly) be involved in singlet oxygen signaling in *E*. *coli*^87^. Our results, however, indicate that intracellularly generated ^1^O_2_ does not result in the activation of the OxyR regulon. The observed differences in the *E*. *coli* expression pattern thus give a first indication that the two tested LOV-FPs represent new blue-light responsive ROS-generators that can be used for studying specific hydrogen peroxide- or singlet oxygen-mediated stress responses in bacteria.

## Discussion

In this study, we investigated the phototoxicity of eleven LOV-FPs in *E*. *coli* and analyzed differences in efficiency and selectivity of ROS formation. We could demonstrate that most of the flavin-binding fluorescent proteins are capable of producing singlet oxygen and hydrogen peroxide to a certain extent. Consequently, the tested LOV-FPs exhibit low to high phototoxicity when applied for light-driven killing of bacterial cells. Basically, the encapsulation of the photosensitizing flavin chromophore by the LOV domain should guarantee that the individual photoinitiated processes are independent of the PS’s surrounding. However, it is clearly not the case, since the specific amino acid sequence of each protein dramatically modulates the properties of the chromophore. In addition, photostability is a key factor in the outcome of aPDT applications. In this regard, the alleged lack of consistency of the photokilling experiments with the ^1^O_2_ production capacity has been rationalized by the photobleaching of the photosensitizing proteins, and a clear correlation between the amount of ^1^O_2_ and the antimicrobial activity is observed when applying low light-doses.

The data presented here demonstrate that the characterized LOV-FPs clearly expand the toolbox of optogenetic sensitizers. Especially, (i) differences in singlet oxygen quantum yields, (ii) selective formation of ^1^O_2_ and H_2_O_2_ as well as (iii) gradual differences in photodynamic inactivation of *E*. *coli* cells render them suitable photosensitizers for a broad range of *in vivo* applications.

For example, since increasing and multiple antibiotic resistances of different Gram-positive and Gram-negative pathogens represent one of the most important therapeutical challenges, alternative agents are urgently needed. Here, genetically encoded PS with high phototoxicities can help to selectively fight against human pathogens, without producing resistances by using them, for instance, as recombinant immunophotosensitizer. The applicability of immonophotosensitizers was already demonstrated for targeted tumor therapy. For example, KillerRed and miniSOG could genetically be fused to the single-chain variable fragment antibody 4D5scFv, which specifically binds to HER2/neu tyrosine kinase receptors. In that way, both immunophotosensitizers exhibited light-mediated cytotoxic effects on HER2/neu-hyperexpressing tumor cells (see^14^ and references therein) - a therapeutic strategy that could easily be transferred to selective aPDI approaches. Alternatively, LOV-based PS can be intracellularly targeted to different compartments where ^1^O_2_- and H_2_O_2_-induced stress can be triggered by light with a high spatio-temporal resolution. This may facilitate the development of new aPDI strategies as well as foster studies regarding ROS-stress thereby amending current methods and therapies with chemical photosensitizers. In future, bacteria that are able to selectively target and proliferate in tumors can be further used for local delivery of tailor-made photosensitizer proteins and subsequent photodynamic treatment as recently demonstrated with KillerRed^88^. Because of the low penetration depth of blue light into mammalian tissues, both aPDI and PDT might be limited to surface exposed pathogens and tumors. However, new surgery techniques nowadays allow delivering light to almost any region of the human body via endoscopes and fiber optics, as for example discussed in^70^.

In contrast, low to moderately phototoxic LOV-PS could be utilized as new optogenetic tools for light-triggered control of bacterial cell growth. This application could be of particular importance e.g. for studying natural microbial communities, which often rely on symbiotic relationships of organisms with unknown biological functions. Here, genetically encoded PS can be used to specifically tag an individual species residing within a community or biofilm thereby opening up new optogenetic strategies to analyze its *in vivo* function.

Finally, we would like to point out that genetically encoded PSs such as miniSOG are shown to be useful tags for analyzing the function of target proteins inside living cells and tissues via light-controlled inhibition (CALI)^14^. This strategy is universally applicable for living cells and tissues, although ROS formation has to be carefully adjusted to avoid off-target effects or even cell death due to high amounts of freely diffusing ROS. Here, the LOV-based optogenetic sensitizers presented in our study exhibiting different ROS formation capabilities again can directly help to find suitable conditions for efficient CALI without harming the surrounding cellular molecules.

Consequently, the alternative LOV-photosensitizers can be applied as a versatile light-responsive biobrick system with adjustable phototoxicities which will be highly beneficial for future optogenetic and biomedical applications.

## Methods

### Construction of LOV-FPs expression vectors

Genes encoding SOPP, CreiLOV and iLOV harboring an *Nde*I and *Xho*I restriction site at the respective 5′- and 3′- end were obtained by commercial gene synthesis (Eurofins Genomics, Ebersberg, Germany). Subsequently, the synthetic DNA fragments were cloned into the *Nde*I and *Xho*I sites of the pET28a vector (Novagen, distributed by Merck KGaA, Darmstadt, Germany). DsFbFP M49I was generated by overlap extension PCR using the pET28a DsFbFP vector DNA^65^ as template and the oligonucleotide primers DsFbFP-up: 5′-CAGCCATATGCGCAGAC-3′, DsFbFP-dn: 5′- GTGCTCGAGTCAGACCGGG-3′, DsFbFP 49I-up: 5′-CAACCCGATTATCTATGTC-3′ and DsFbFP 49I-dn: 5′-GACATAGATAATCGGGTTG-3′. The resulting final PCR fragments were hydrolyzed with *Nde*I and *Xho*I and ligated into the respective sites of the pET28a vector. DNA cloning was conducted using the *Escherichia coli* strain DH5α^89^ and DNA plasmid isolations from bacterial cells were performed using the commercial innuPREP Plasmid Mini Kit (Analytik Jena, Jena, Germany), as described by the manufacturer. All final vector constructs were verified by DNA-sequencing (Eurofins Genomics, Ebersberg, Germany).

### *Escherichia coli*-based *in vivo* phototoxicity assays

Time-resolved, qualitative comparison of phototoxic effect caused by eleven FbFPs (Table 1) on *E*. *coli* BL21(DE3) (Novagen, distributed by Merck KGaA, Darmstadt, Germany) was carried out by plate spot assay. *E*. *coli* cells harboring the respective LOV-FP expression vectors were cultivated in 100 ml flasks at 37 °C. Three hours after induction of LOV-FP expression (addition of 0.4 mM IPTG), samples were taken out of the expression cultures and diluted to a final cell density (OD_580_ = 0.025) in PBS-Buffer (140 mM NaCl; 12.5 mM Na_2_HPO_4_; 2.7 mM KCl; 1.8 mM KH_2_PO_4_, pH 7.4). The cell suspension was transferred into a macro cuvette and placed directly on top of a blue-light emitting LED (LUXEON Rebel XLML PR01 0425; λ = 448 nm; 130 mW cm^−^²). At given time points (0 to 120 sec) 3 µl-aliquots were taken out of the illuminated cell solutions and dropped on an LB agar plate, containing 0.2% lactose. A culture of *E*. *coli* cells carrying the empty vector pET28a as well as non-irradiated samples of each LOV-FP expression culture were used for appropriate control experiments. Subsequently, agar plates were incubated at 37 °C overnight. To distinguish between fluorescing and non-fluorescing colonies, agar plates were finally photographed under UV-light (λ = 365 nm) illumination. Phototoxicity of LOV-FPs is indicated by an impaired cell growth and only fluorescent colonies were taken into account, as non- fluorescent colonies represent cells that did not express the protein at the time of illumination due to population heterogeneity.

To quantitatively determine the cell viability rate of *E*. *coli* expressing different phototoxic LOV-FPs the colony forming capacity was measured in dependence of exposure time light intensity. *E*. *coli* BL21 (DE3) cells harboring the respective expression vectors pET28a-phiLOV2.1, pET28a-DsFbFP, pET28a-DsFbFP M49I and pET28a-EcFbFP, respectively, were cultivated in 100 mL flasks at 37 °C. Three hours after induction with 0.4 mM IPTG, the fluorescence spectra of the LOV-based FbFPs were measured in whole cell extracts that have been resuspended in PBS buffer (pH 7.4). To minimize the influence of different growth rates in the expression cultures, all spectra were normalized to OD_580_ = 1. The fluorescence emission spectra were measured using a microplate reader (Infinite M1000 Pro, Tecan Group LTD., Maennedorf, Switzerland). For determination of colony forming units (CFU) before and after illumination, the cultures were diluted to a final OD_580_ of 0.1 in PBS buffer (pH 7.4). The cell suspension was transferred into a macro cuvette and illuminated with different intensities (90 mW cm^−2^ and 130 mW cm^−2^) of a blue light emitting LED (LUXEON Rebel XLML PR01 0425; λ_max_ = 448 nm). To reach an illumination intensity of 10 mW cm^−2^ the samples were transferred into a 48-well microtiter plate (FlowerPlate; m2p-laps GmbH,Aachen,Germany) and illuminated with blue light (λ_max_ = 447 nm, ~10 mW cm^−^²) in a microbioreactor (BioLector; m2p-laps GmbH, Aachen, Germany). At given time points (0 to 180 s), 100 µL of the irradiated cell solutions were taken out of the respective illumination setup and diluted to predefined cell densities in LB-media (here, an appropriate dilution was chosen which finally results in approximately 100–200 colonies on agar plates). From these samples, 100 µL were plated on LB agar plates and incubated at 37 °C overnight in the dark. Additionally, a non-irradiated dark-treated sample of each FbFP expression culture was used as a control.

Quantitative analysis of light-induced cell-death was conducted using propidium iodide (PI), a fluorescent dye that selectively enters dead cells and develops a specific fluorescence signal with λ_max_ at 617 nm when excited with yellow light (535 nm) after intercalation into DNA^68^. To comparatively characterize phototoxicities of cytoplasmic LOV-FPs in *E*. *coli*, chemo-competent *E*. *coli* BL21(DE3) cells were transformed with recombinant pET28a vectors allowing the expression of the respective fluorescent reporter genes. Therefore, *E*. *coli* expression cultures were inoculated with an optical density (OD_580_) of 0.1 in Terrific Broth (Carl Roth, Karlsruhe, Germany) auto-induction media, supplemented with 0.2% lactose, 0.03% glucose and 50 µg ml^−1^ Kanamycin. Cell cultivations (culture volume: 800 µl) were performed in microtiter plate (FlowerPlate) using the BioLector (m2p-labs GmbH, Baesweiler, Germany), for six hours at 37 °C under continuous shaking (1500 rpm). Subsequently, cultures were harvested via centrifugation, washed with PBS (pH 7.4) and finally resuspended in 1300 µL assay buffer (PBS pH 7.4, 100 µM EDTA, 5 µM PI, Sigma Aldrich) with a final cell density of OD_580_ = 0.5. These samples were subsequently transferred to a fresh FlowerPlate and placed into the BioLector with a LED blue light source for homogeneous illumination of the entire flower plate. To ensure optimal oxygen supply for photosensitizing, *E*. *coli* cells were illuminated with blue light (λ_max_ = 447 nm, ~10 mW cm^−2^) under continuous shaking (1100 rpm, 30 °C). At several time points, 100 µl samples were taken and analyzed regarding their LOV-FP-mediated fluorescence intensity (excitation at 450 nm, emission at 495 nm) and PI fluorescence (excitation at 535 nm, emission at 617 nm) using a microplate reader (Infinite M1000 Pro, Tecan Group LTD., Maennedorf, Switzerland). To be able to evaluate LOV-FP-mediated phototoxicity regardless of its respective expression efficiency in *E*. *coli*, the data was normalized using equation 1, where I_n_ is the normalized PI fluorescence intensity, I_raw_ the raw PI fluorescence, I_FP_ the LOV-FP-fluorescence intensity of the cell culture before blue-light illumination, *Φ*_F_ the fluorescence quantum yield and ε the molar extinction coefficient of the respective LOV-FP (see Table 1):

1 $${I}_{n}=\frac{{I}_{raw}}{(\frac{{I}_{FP}}{{\Phi }_{F}\times \varepsilon })}$$

### Heterologous expression, purification and spectral characterization of LOV-based FPs

Expression and purification of all tested LOV-based fluorescent proteins (Table 1) was performed as described before^65^, except that the isolated proteins were stored in phosphate buffered saline (PBS), pH 7.4. Furthermore, spectroscopic and photophysical analysis of CreiLOV, DsFbFP M49I, iLOV and SOPP, including the determination of fluorescence quantum yields and extinction coefficient followed the descriptions in the above mentioned publication.

### Spectroscopic determination of singlet oxygen quantum yields of isolated LOV-FPs

Direct detection of ^1^O_2_ phosphorescence at 1275 nm was carried out using a customized PicoQuant Fluotime 200 lifetime system and an AO-Z-473 solid state AOM Q-switched laser (Changchun New Industries Optoelectronics Technology Co., China), used for excitation at 473 nm (<1.5 mW average power) and working at 1.5 kHz repetition rate. Further details on the instrumentation and methods used for ^1^O_2_ detection are described elsewhere^45,90^. The time-resolved emission decays were analyzed by fitting *Eq*. *2* to the data using GraphPad Prism 5.

2 $${S}_{(t)}={S}_{(0)}\frac{{\tau }_{{\rm{\Delta }}}}{{\tau }_{{\rm{\Delta }}}-{\tau }_{{\rm{T}}}}({e}^{\frac{-t}{{\tau }_{{\rm{\Delta }}}}}-{e}^{\frac{-t}{{{\rm{\tau }}}_{{\rm{T}}}}})$$

*τ*_T_ and *τ*_Δ_ are the lifetimes of the photosensitizer triplet state and of ^1^O_2_, respectively and *S*_(0)_ is a quantity proportional to *Φ*_Δ_.

*Φ*_Δ_ was determined by comparing the *S*_(0)_ values of optically-matched solutions of the corresponding flavoproteins and flavin mononucleotide (FMN) at 473 nm (Eq. 3)^91^.

3 $${{\rm{\varphi }}}_{{\rm{\Delta }},flavoprotein}=\frac{{S}_{(0)flavoprotein}}{{S}_{(0)FMN}}{{\rm{\varphi }}}_{{\rm{\Delta }},FMN}$$

FMN was taken as reference photosensitizer with *Φ*_Δ_ = 0.51 in PBS^92^ and 0.57 in dPBS^45^.

### Determination of superoxide-anion and hydrogen peroxide formation of purified LOV-FPs

For the determination of LOV-FP-mediated O_2_^−^/H_2_O_2_ production the commercial Amplex® Red Hydrogen Peroxide/Peroxidase Assay Kit (Molecular Probes, Invitrogen) was used. The analysis was performed according to the manufacturer’s manual with purified LOV-FPs adjusted to a final absorbance of 0.05 at λ  = 450 nm. To determine entire type I-dependent ROS formation, 4 U ml^−1^ superoxide dismutase (Sigma Aldrich) was supplemented to the reaction buffer to catalyze the conversion of superoxide anions to H_2_O_2_. Sample irradiation was conducted in microtiter plates (FlowerPlates) within a microbioreactor (BioLector, m2p-labs GmbH, Baesweiler, Germany) at 1100 rpm and 30 °C with a LED blue light source emitting light with λ_max_ at 447 nm and an intensity of ~10 mW cm^−2^. Fluorescence intensities of resorufin (the product of the Amplex Red reaction) were determined in a microplate reader (Infinite® M1000 Pro, Tecan Group LTD., Maennedorf, Switzerland) with an excitation wavelength of λ = 571 nm and an emission at λ = at 585 nm. To determine final H_2_O_2_ concentrations after LOV-FP illumination, the measured resorufin fluorescence intensities were subsequently compared with an H_2_O_2_ calibration curve (0 to 20 µM H_2_O_2_).

### Analysis of LOV-FP-mediated stress response in *E*. *coli*

To analyze differences in global oxidative stress response in *E*. *coli* that were induced by Pp2FbFP and DsFbFP M49I, a DNA microarray-based analysis was conducted. *E*. *coli* cells expressing the respective photosensitizers were cultivated in LB-media, supplemented with 50 µg ml^−1^ kanamycin (25 ml culture volume in 500 ml Erlenmeyer flasks, start OD_580_ = 0.05). The cultures were incubated at 37 °C and under continuous shaking (130 rpm) in the dark. LOV-FP expression was induced by addition of 0.4 mM IPTG during the logarithmic growth phase (~OD_580_ = 0.7). Three hours after induction, cultures were divided into two test cultures with a volume of 25 ml in 500 ml Erlenmeyer flasks and an OD_580_ of 0.5. For light treatment one of these cultures was placed between two blue-light emitting LED-panels (λ = 462 nm, 100 mW cm^−2^, Insta Elektro, Lüdenscheid, Germany, panel distance: 30 cm), whereas the second culture was kept in the dark. To ensure optimal oxygen supply of cells during irradiation process, cultures were supplemented with stirring bars and placed on magnetic stirrers. Since the selectivity and efficiency of light-driven ROS formation strongly vary between those proteins, we first evaluated different exposure times (5, 10, 15 and 30 minutes) for full induction of LOV-FP-mediated stress-response in *E*. *coli*. To this end, samples containing an OD_580_ of 3 were harvested after light treatment by centrifugation and the cell pellets were shock frozen in liquid nitrogen and subsequently stored at −80 °C. RNA isolation, cDNA synthesis, microarray hybridization and data analysis were performed as described before^93^ except the utilization of DNA-microarrays carrying probes for the genes of *E*. *coli* MG1655 strain. Detailed transcriptome analyses of light-induced stress-response based on independent biological triplicates were finally carried out under best illumination conditions (i.e. Pp2FbFP: 5 min, DsFbFP M49I: 15 min) as described above.

## Electronic supplementary material

Supplemental material

## Data Availability

The datasets generated during and/or analyzed during the current study are available from the corresponding authors on reasonable request. The microarray data generated and analyzed in this study are accessible in NCBI’s Gene Expression Omnibus through accession number GSE110168.
