# Supplementary material for: An optogenetic toolbox of LOV-based photosensitizers for light-driven killing of bacteria
Source: Sci Rep. 2018 Oct 9;8:15021. doi: 10.1038/s41598-018-33291-4 (PMC6177443; doi:10.1038/s41598-018-33291-4)
Supplement: Supplementary file 1 — Supplemental material [file 41598_2018_33291_MOESM1_ESM.pdf]

Supplemental material

An optogenetic toolbox of LOV-based photosensitizers for light-driven killing of bacteria

Authors

Stephan Endres[1,2,§], Marcus Wingen[1,§], Joaquim Torra[3,§], Rubén Ruiz-González[3], Tino Polen[4], Gabriela Bosio[5,6], Nora Lisa Bitzenhofer[1], Fabienne Hilgers[1], Thomas Gensch[6], Santi Nonell[3,*], Karl-Erich Jaeger[1,4], Thomas Drepper[1,*]

Affiliations

[1] Institute of Molecular Enzyme Technology, Heinrich-Heine-University Düsseldorf, Forschungszentrum Jülich GmbH, Jülich, Germany

[2] m2p-labs GmbH, Baesweiler, Germany

[3]Institut Químic de Sarrià, Universitat Ramon Llull, Barcelona, Spain

[4] Institute of Bio- and Geosciences, IBG-1: Biotechnology, Forschungszentrum Jülich GmbH, Jülich, Germany

[5] Instituto de Investigaciones Teóricas y Aplicadas, Universidad Nacional de La Plata, Argentina.

[6] Institute of Complex Systems, ICS-4: Cellular Biophysics, Forschungszentrum Jülich GmbH, Jülich, Germany

[§] contributed equally to this work
* Corresponding authors
E-Mail: t.drepper@fz-juelich.de
santi.nonell@iqs.url.edu

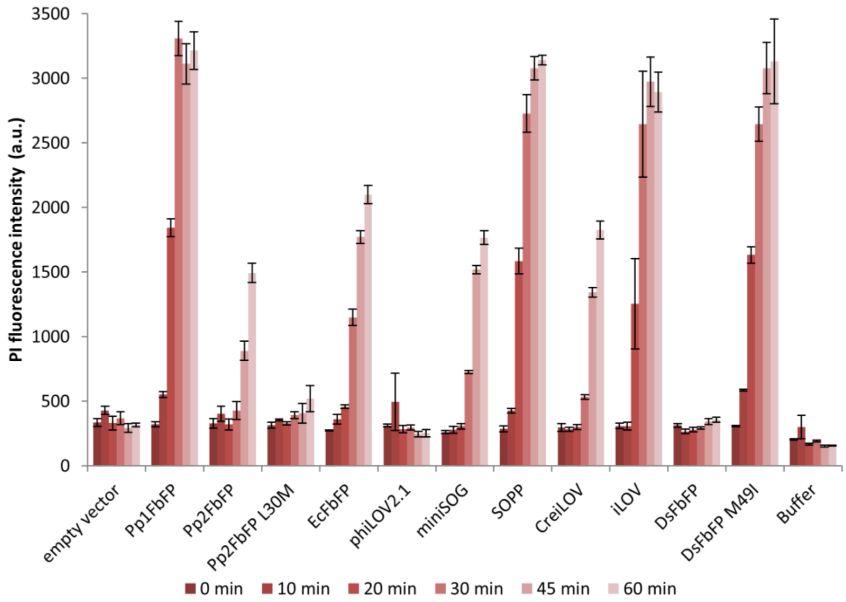

Supplemental figure S1. Raw data of the analysis of LOV-FP phototoxicity using propidium iodide as quantitative marker for dead *E. coli* cells. The bars show the raw PI fluorescence intensity (λ_{ex} = 535 nm, λ_{em} = 617 nm) over 60 minutes. Data represents the mean values of three independent experiments and the error bars the calculated standard deviations.

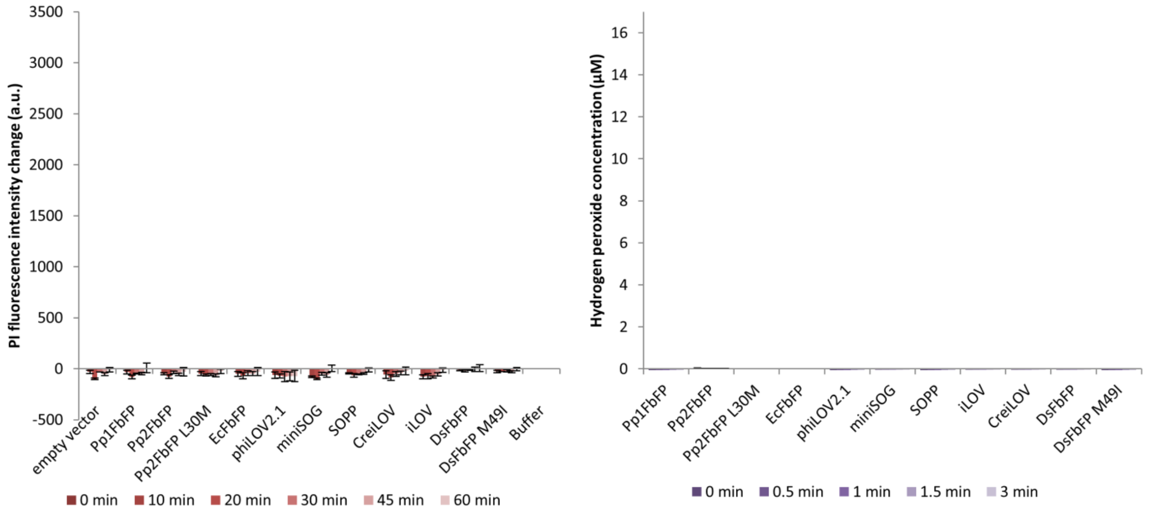

Supplemental figure S2. Control experiments of the PI assay and Amplex Red assay in the dark. A: PI toxicity assay in the dark. The data show the change in PI fluorescence intensity compared to that at t = 0. No significant changes could be observed. B: Amplex Red assay in the dark. The bars show the change in hydrogen peroxide concentration at different exposure times in the dark for all tested photosensitizes. No detectable H_2O_2 production was observed. The data represent the mean values of three independent experiments and the error bars the calculated standard deviations.

Supplemental figure S3. Analysis of heterogeneity of LOV-FP-mediated cell death at the single cell level.

The experiments devoted to study LOV-FP induced phototoxic effects in single *E. coli* cells via PI fluorescence detection were performed on a spinning disk confocal fluorescence microscope as previously described (Oreopoulos and Browne, 2014) First, *E. coli* BL21 (DE3) cells were transformed with the respective LOV-FP expression vector or empty vector. The transformed cells were cultivated overnight in TB media at 37 °C and subsequently induced in the stationary growth phase with 0.4 mM IPTG. The cells were harvested 3 h after induction, resuspended in PBS buffer (pH 7.4) and then immobilized on glass bottom 35 mm Petri dishes (ibidi, Martinsried, Germany), previously treated with Poly-L-Lysine (PLL) (0.1 mg.mL−1) (1 mL for 5 min). Samples were then placed directly inside a stage top incubator (Okolab S.R.L., Pozzuoli, Italy; environmental parameters set to 37 °C and 85% humidity in air atmosphere) on the stage of an inverted microscope (Ti-E, Nikon Europe BV, Düsseldorf, Germany). The inverted microscope was used as a spinning disk confocal microscope as described by Oreopoulos and Browne (2014) (ACALBFI, Groeben, Germany) with a spinning disk unit (CSU-W1; Yokogawa Electric Corporation, Tokyo, Japan) as the central part (more details in Rivas Aiello *et al.* 2018). Bright field images, as well as spinning disk confocal fluorescence images with excitation at 488 nm and 561 nm were taken with a 100X magnification oil immersion objective (plan apo chromat, NA=1.40, Nikon). Appropriate dichroic beam splitter and bandpass filters in the Optosplit unit allowed the detection of LOV-FP fluorescence (λ_{exc} = 488 nm (laser); λ_{obs} = 500 - 530 nm) or PI fluorescence (λ_{exc} = 561 nm (laser); λ_{obs} = 575 - 630 nm), respectively. First, *E. coli* cells were exposed to a buffer containing 1.5 μM PI for 5 to10 minutes and transmission (bright field; Supplemental Fig. S3A I, S3B I, S3C I), LOV-FP fluorescence (Supplemental Fig. S3A II, S3B II, S3C II) and PI fluorescence images (Supplemental Fig. S3A III, 3B III, 3C III) were acquired. Irradiation for photodynamic action was performed with fluorescence excitation lamp of the microscope (465-495 nm; 32 μW in an illuminated spot with 200 μm diameter (i.e., 102 mW cm^{-2})) and PI fluorescence images were recorded at several time points and a total illumination time of 10 minutes.

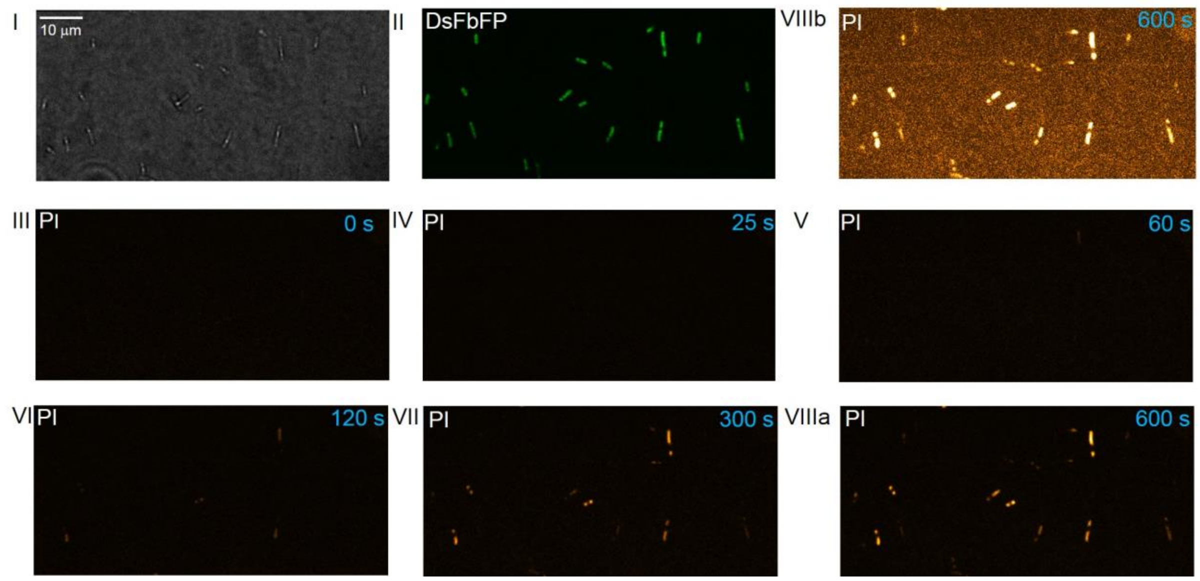

Supplemental figure S3A. Phototoxicity of DsFbFP. Images of DsFbFP expressing *E. coli* cells before blue light irradiation (I, bright field; II, DsFbFP fluorescence emission; III PI fluorescence emission) and PI emission images after different blue light exposures (460-490 nm; P=102 mW cm^{-2}) (IV – VIII). Fluorescence intensity scale for image II is 135-4135 and for III-VIIIa amounts to 135 – 10135 (II: λ_{exc}=488 nm, P=95 mW/cm^2, t=100 ms; III-VIIIb: λ_{exc}=561 nm, P=140 mW cm^{-2}, t=50 ms). VIIIb is the same image as VIIIa but with a ten-fold smaller scale (135-1135) for better visualization of phototoxic effect in all DsFbFP-expressing *E. coli* cells.

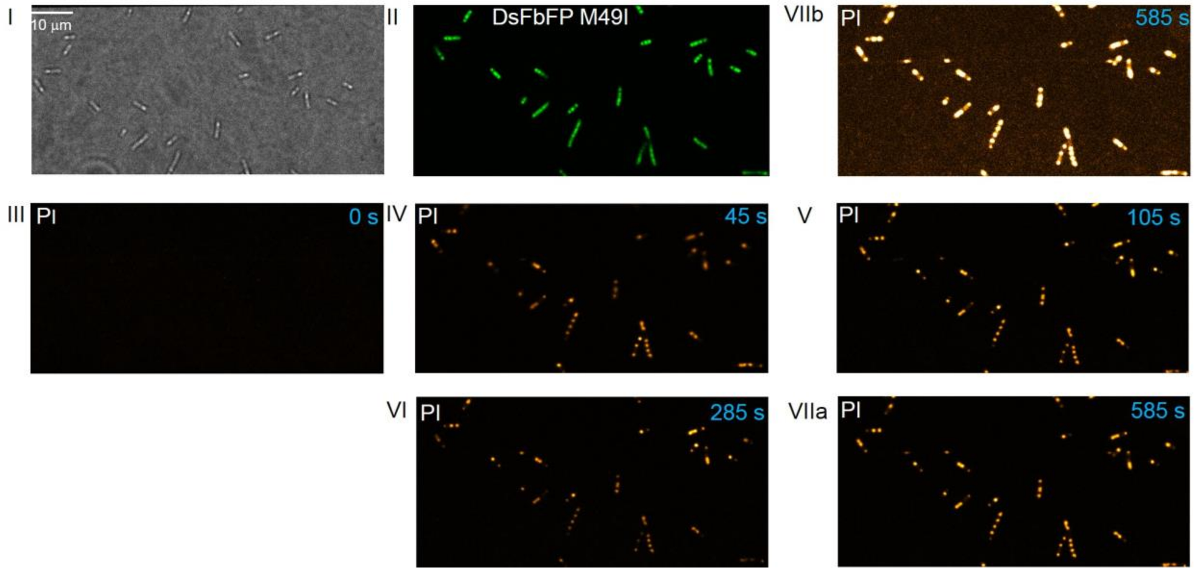

Supplemental figure S3B. Phototoxicity of DsFbFP M49I. Images of DsFbFP M49I expressing *E. coli* cells before blue light irradiation (I, bright field; II, DsFbFP fluorescence emission; III PI fluorescence emission) and PI emission images after different blue light exposures (460-490 nm; P=102 mW cm^{-2}) (IV – VIII). Fluorescence intensity scale for II is 135-4135 and for III-VIIa amounts to 135 – 28135 (II: λ_{exc}=488 nm, P=95 mW/cm^2, t=100 ms; III-VIIb: λ_{exc}=561 nm, P=140 mW cm^{-2}, t=50 ms). VIIb is the same image as VIIa but with a tenfold smaller scale (135-2935) for better visualization of phototoxic effect in all DsFbFP M49I-expressing *E. coli* cells. Please note that the bright field image (I) was shifted by accident (~5 µm to the bottom and 2 µm to the left), compared to the fluorescence images (II - VII).

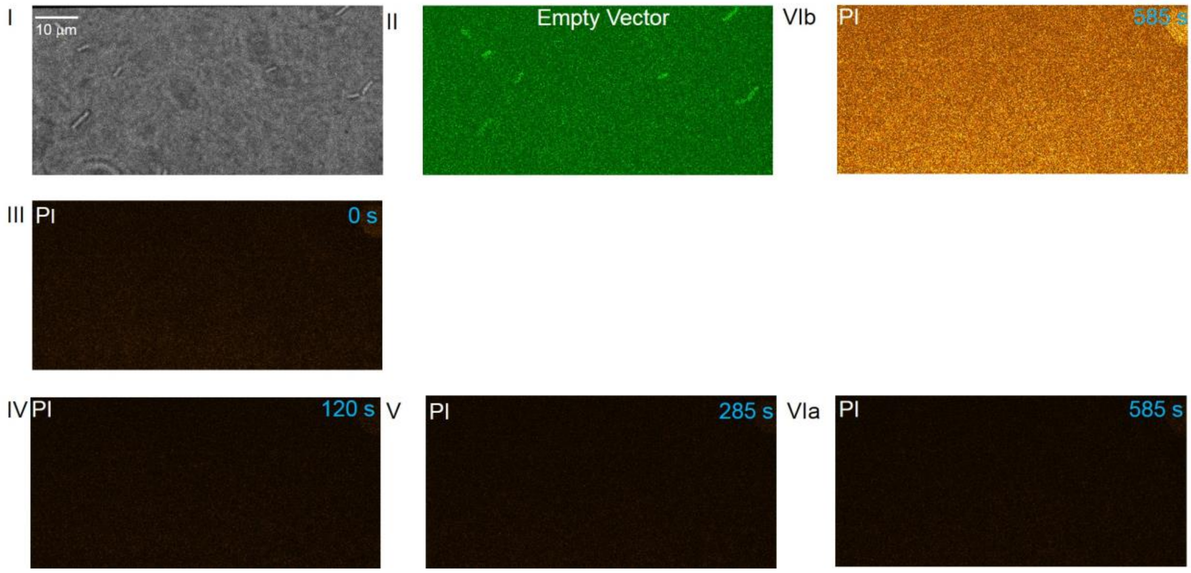

Supplemental figure S3C. Phototoxicity control experiment with *E. coli* cells carrying the empty expression vector. Images of *E. coli* cells before blue light irradiation (I, bright field; II, autofluorescence of endogenous flavins; III PI fluorescence emission) and PI emission images after different blue light exposures (460-490 nm; P=102 mW cm^{-2}) (IV – VI). Fluorescence intensity scale for II is 135-535 and for III-VIIa amounts to 135 – 4135 (II: λ_{exc}=488 nm, P=95 mW/cm^2, t=100 ms; III-VIIb: λ_{exc}=561 nm, P=140 mW cm^{-2}, t=50 ms). VIIb is the same image as VIIa but with a tenfold smaller scale (135-535) for better visualization of the absence of phototoxic effect in all *E. coli* cells transfected with empty vector.

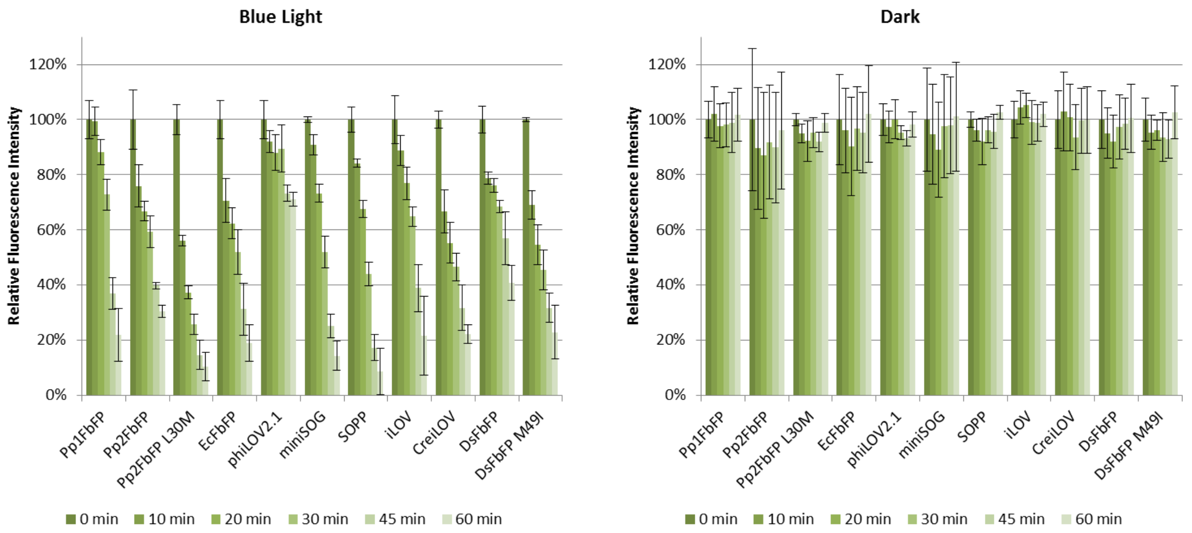

Supplemental figure S4. *In vivo* photobleaching of LOV-FP fluorescence intensities in dependence to blue light irradiation. The bars indicate the normalized LOV-PS fluorescence intensity (λ_{ex} = 450 nm, λ_{em} = 495 nm) for the light- (left) and control experiment (right). For this study, the same samples have been used as for the *in vivo* PI assay that is shown in Fig. 3. Prolonged irradiation with blue light (λ_{max} = 447 nm, ~ 10 mW cm^{-2}) led to a decreasing activity of all tested variants, whereas explicit differences were found. Some variants, like phiLOV2.1 showed only weak photobleaching whereas Pp2FbFP L30M appeared notably sensitive. As expected, no significant changes within the LOV-PS fluorescence activity was observed in the control experiment. The given data represents the mean values of three independent experiments and the error bars the calculated standard deviations.

References

Rivas Aiello, M., Castrogiovanni, D., Parisi, J., Azcárate, J., García Einschlag, F., Gensch, T., Bosio, G., Martire, D. Photodynamic Therapy in HeLa Cells Incubated with Riboflavin and Pectin-coated Silver Nanoparticles. *Photochem Photobiol* accepted (2018)

Oreopoulos, J., Berman, R., Browne, M. Spinning-disk confocal microscopy: present technology and future trends. *Meth Cell Biol* **123**, 153-175 (2014).
